# Evaluating ZEBRA Battery Module under the Peak-Shaving Duty Cycles

**DOI:** 10.3390/ma14092280

**Published:** 2021-04-28

**Authors:** Nimat Shamim, Edwin C. Thomsen, Vilayanur V. Viswanathan, David M. Reed, Vincent L. Sprenkle, Guosheng Li

**Affiliations:** Battery Materials & System Group, Pacific Northwest National Laboratory, Richland, WA 99352, USA; nimat.shamim@pnnl.gov (N.S.); Edwin.Thomsen@pnnl.gov (E.C.T.); Vilayanur.Viswanathan@pnnl.gov (V.V.V.); David.Reed@pnnl.gov (D.M.R.); Vincent.Sprenkle@pnnl.gov (V.L.S.)

**Keywords:** sodium nickel chloride battery, ZEBRA battery, grid energy storage, peak shaving duty cycle, long-term cycling

## Abstract

With the recent rapid increase in demand for reliable, long-cycle life, and safe battery technologies for large-scale energy-storage applications, a battery module based on ZEBRA battery chemistry is extensively evaluated for its application in peak shaving duty cycles. First, this module is tested with a full capacity cycle consisting of a charging process (factory default) and a discharging process with a current of 40 A. The battery energy efficiency (discharge vs. charge) is about 90%, and the overall energy efficiency is 80.9%, which includes the auxiliary power used to run the battery management system electronics and self-heating to maintain the module operating temperature (265 °C). Generally, because of the increased self-heating during the holding times that exist for the peak shaving duty cycles, the overall module efficiency decreases slightly for the peak-shaving duty cycles (70.7–71.8%) compared to the full-capacity duty cycle. With a 6 h, peak-shaving duty cycle, the overall energy efficiency increases from 71.8% for 7.5 kWh energy utilization to 74.1% for 8.5 kWh. We conducted long-term cycling tests of the module at a 6 h, peak-shaving duty cycle with 7.5 kWh energy utilization, and the module exhibited a capacity degradation rate of 0.0046%/cycle over 150 cycles (>150 days).

## 1. Introduction

The continuing growth of electricity generation using renewable resources, such as wind and solar power, has stimulated the development of low-cost and safe energy-storage devices for reliable grid operations [1,2]. Conventional lithium (Li)-ion batteries (LIBs) have been used widely for decades in portable electronics and electrical vehicle applications. However, because of materials scarcity and intrinsic safety concerns associated with its high flammability, the application LIBs for large-scale energy storage is still under debate. To accelerate broader market penetration of rechargeable batteries in grid applications, battery research and development has been greatly focused on finding cost-effective and viable alternatives to LIBs [3,4].

Among various sodium (Na)-based rechargeable batteries, sodium-metal halide (Na-MH or ZEBRA) batteries use low cost and abundant Na, nickel (Ni), and iron (Fe) as the main battery constituents and also offer superior battery safety and durability, thereby providing great potential for various grid applications [5,6,7]. The ZEBRA battery using Ni/Fe cathodes is the most popular redox chemistry among the vast majority of Na-MH batteries including Na-NiCl_2_, Na-FeCl_2_, Na-ZnCl_2_, and Na-Al batteries reported in the past [8,9,10,11,12,13,14,15,16,17,18], and the overall cell reaction of ZEBRA battery can be described as follows [19,20]:

2Na + MCl_2_ (Charge) ←→ 2NaCl + M (Discharge), (1)

where M is Ni, Fe with E_0_ = 2.58 V for Ni, and 2.35 V for Fe, respectively. The tubular or clover shape β″-Al_2_O_3_ solid electrolyte (BASE) tube has been used for ZEBRA batteries as a key component to facilitate Na^+^ ion transportation but stop material cross-over between the cathode and anode sides [20]. It should be noted that the ZEBRA battery is typically assembled in the discharge state using a mixture of nickel/iron powder, sodium chloride, and sodium tetra chloroaluminate (NaAlCl_4_), and a few additives [20]. This approach greatly helps to avoid issues associated with handling anhydrous metal chlorides and pyrophoric sodium metal if the cells are assembled in the charged state. During the initial charging process, Na^+^ ions from NaCl move through a liquid electrolyte (NaAlCl_4_) and the BASE tubes to form molten Na at the anode, while metal chlorides (NiCl_2_ and FeCl_2_) are formed simultaneously at the cathode. During the subsequent discharging process, Na^+^ ions return to form NaCl at the cathode side where the reduction in metal chloride to metal take place. Conventional ZEBRA batteries typically are operated at a temperature between 265 and 350 °C to achieve a high Na^+^ ion conductivity of BASE and faster cell redox chemistry; however, the operating temperature can be as low as 157 °C, which is determined by the melting temperature of the NaAlCl_4_ used as a secondary liquid electrolyte in ZEBRA batteries to facilitate the movement of Na^+^ ions between the BASE and solid cathode materials. Recently, Na-MH batteries operated at lower temperatures (<200 °C) have been actively investigated [21,22,23]. This new battery technology has shown promising performance by slowing battery capacity degradation via grain growth suppression and possible cost reduction by adopting polymer seals to replace complicated battery sealing technologies (such as thermocompression bonding, glass seals, etc.) generally used at higher operating temperatures [7,24]. One of most important advantages of a ZEBRA battery compared to its analogous high-temperature Na-S battery technology is that metal halide-based cathodes are less corrosive and more stable than molten polysulfide cathodes in a Na-S battery at high temperatures. Additionally, the use of NaAlCl_4_ in the ZEBRA battery provides superior battery safety when BASE tubes in the ZEBRA batteries mechanically fail [25].

Since its invention in the early 1980s [26], large-scale production of ZEBRA battery technologies has been pursued by various entities including BETA Research Development, AEG Anglo Batteries, MES-DEA, etc. Currently, the FzSoNick Group, a part of FIAMM Energy Technology, operates a commercial ZEBRA battery production facility. It is worth mentioning that before the rise of LIB technologies, ZEBRA battery technology initially was developed for full electric vehicle and hybrid electric vehicle applications because the battery pack can provide high energy density (~100 Wh/kg) [25,27]. Additionally, because of its long life at the fully charged state and ability to operate in a broad range of environmental conditions (i.e., hot or cold weather), the ZEBRA battery is particularly suitable for applications in the telecommunication industry to provide standby power for facilities located at remote areas where conventional rechargeable batteries may encounter problems due to extreme climate conditions [7]. Experimental and extended modeling studies of ZEBRA battery application for telecom application have been reported recently [28]. Despite the higher price of ZEBRA batteries compared to that of conventional LIBs, the ZEBRA technology still is considered to be one of the most important battery technologies for large-scale energy storage applications because of its longevity and superior safety. More advanced Na-MH battery technologies with improved characteristics, including the ability to operate at lower temperatures, cost-effective cell architectures, and low-cost cathode materials, could lead to substantial cost reductions for the ZEBRA technology, thereby promoting use in large-scale energy storage applications.

In the work described in this paper, we study a ZEBRA battery module (48TL200) from FzSoNick to gain an understanding of its performance when operated in peak-shaving mode, which is an important grid energy-storage application. We test the module under three different duty cycles that have different discharge times of 2, 4, and 6 h. A detailed analysis of the battery module has been conducted to evaluate temperature trends, energy efficiency, and capacity degradation under peak-shaving duty cycles. We believe the results of our studies provide valuable understanding of ZEBRA battery technologies for grid energy-storage applications.

## 2. Experiment Methods

### 2.1. Module Specification

The 48TL200 battery module was purchased from FZSoNick and tested without further modification. Representative technical specifications are summarized in Table 1.

The module capacity is rated at 200 Ah (40 Ah × 5), because it has five battery strings connected in parallel, and each string is composed of 20 ZEBRA cells, each with a capacity of 40 Ah. The nominal voltage (48 V) of the module comes from 20-unit cells (~2.4 V of the discharge voltage for the single cell) in a series configuration for each string. The total energy of the module is rated at 9.6 kWh, but this value can vary slightly depending on the operating conditions (such as discharging current, cut-off voltage, etc.). Because of the intrinsic feature of ZEBRA battery chemistry, the module requires a minimum internal temperature of 265 °C and comes with a built-in internal heating system to maintain the appropriate operating temperature. Despite the high operating temperature, the surface temperature of the module enclosure is only 10 to 15 °C above ambient temperature due to the thermal insulation in the module. An integrated battery management system (BMS) of the module regulates the charging current and charging voltage to the optimal level. The BMS provides battery safety by continuously monitoring all the battery parameters such as voltages, currents, temperatures, insulation levels and by calculating the battery state-of-the-charge (SOC) to avoid overcharge or over discharge. For the initial activation process, the BMS starts the warm-up process without connecting the battery module to the direct current (DC) Bus (the internal power switch is still open), if the internal temperature is below 265 °C. Once the target temperature is reached, the main contactor closes to connect the battery module to the DC power supply, and the module then is ready for the testing.

### 2.2. Module Full-Capacity Testing

The integrated BMS of the 48TL200 is designed to charge the module at a constant current (CC) of 40 A until the module voltage reaches 53.4 V followed by a constant voltage (CV) charge at 53.4 V until full capacity (200 Ah) is reached. In contrast to the charging process, the BMS allows more flexible discharge protocols, such as CC or constant power, for the module. The module testing was performed using a NH Research Inc. (NHR, Irvine, CA, USA) battery pack test system (9200 series, model 4912), which has an output range of the voltage and current up to 120 V and 200 A, respectively. Briefly, for the charge process, the NHR system applies on average 56 V (ranging from 54–59 V) DC to the module, and the BMS of the module, which is equipped with an integrated charge regulator (DCDC step-down converter), regulates the charging current and voltage to the appropriate levels to follow the charging process consisting of the CC and CV processes mentioned above. The discharging process for the initial module testing was done at a CC (40 A) with a cut-off voltage of 44.0 V.

### 2.3. Peak Shaving Testing Protocols

The peak-shaving (PS) test based on PS duty cycles in literature is designed to demonstrate the capability of the module while it is tested at various energy levels. The total duration of one peak-shaving cycle consists of one charge cycle, one discharge cycle, and two hold durations, and the hold durations are essential to demonstrate a more realistic PS application and bring the total PS duty cycle to a 24 h period [29]. The battery module is discharged at different power levels for various durations to replicate peak-shaving schedules. For all tests, the module is charged to the full capacity by following the charge process mentioned above (CC + CV). For instance, the module is tested for 6, 4, and 2 h discharge at a total energy of 7.5 kWh with corresponding discharge powers at 1.25, 1.875, and 3.75 kW, respectively. The hold times after the 6, 4, and 2 h discharge tests are 3, 4, and 5 h. The hold time after the charge process varies slightly to compensate for the time variation from the charge process, which is generally close to 12 h. The module is also tested for different discharge energy levels, such as 8.0 and 8.5 kWh. It is worth mentioning that the electric power for the DC bus is supplied by the battery module except during the heating-up and charging periods. During those periods, power is provided by the NHR system. This means that the DC bus consumes electricity from the battery module to maintain the operating temperature during the holding and discharging periods.

### 2.4. Long-Term Testing Protocol

Long-term peak shaving testing of the module is done by using 6 h peak shaving protocol at a discharge energy of 7.5kWh. Each complete cycle, consisting of four steps, is designed to take 24 h. First, the module is charged to full capacity using the default charging process (CC + CV). The charge step takes at least 12 h and is deemed to be complete if the BMS reports the SOC to be 200 Ah (100%). If the SOC reaches 100% in less than 12 h, then the module floats until 12 h has elapsed. If the SOC has not reached 100% capacity within 12 h, then the charging process can continue for up to an additional 1 h. Second, the module rests at an open circuit. During the rest step (end-of-charge (EOC)-hold), the module provides its own power to maintain temperature and the BMS. This step lasts up to 3 h; if the charge step took longer than 12 h, then the EOC-hold is adjusted to keep 15 h for the charge and EOC-hold processes combined. Third, the module is discharged at a constant power of 1.25 kW for 6 h. Fourth, there is another rest step (end-of-discharge (EOD)-hold) of 3 h after the discharge. 

## 3. Results and Discussion

**Figure 1:**
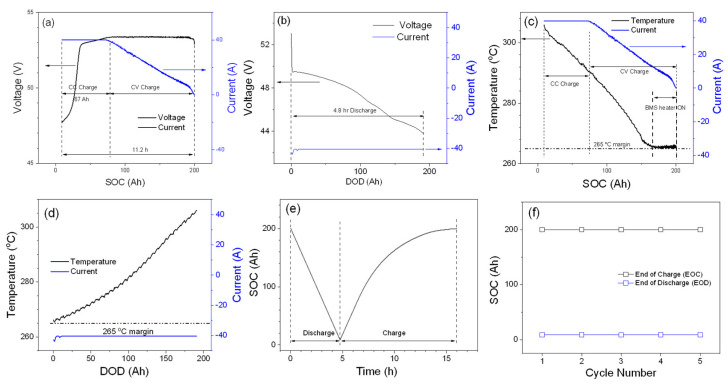
Module performance of full-capacity testing. (**a**,**b**) Voltage and current profile for the charge and dis-charge. (**c**,**d**) Temperature and current profiles of the charge and discharge pro-cesses. (**e**) SOC vs. time. (**f**) SOC at EOC and EOD.

The 48TL200 module was initially tested according to the manufacturer-recommended parameters, and its performance is shown in Figure 1.

Charge voltage and current graphs vs. the SOC of the module are plotted in Figure 1a. As described in the experimental section, a CC charge is done at 40 A until the voltage of the module reaches 53.4 V, and then a CV charge at 53.4 V until the full capacity (SOC at 200 Ah) is achieved. The charged capacity via CC mode is about 67 Ah (~1 h and 40 min), and the total charge time including CC and CV is about 11.2 h for this module. As shown in Figure 1b, the discharge current is fixed at 40 A until the module voltage reaches 44 V, and the battery module is discharged to the depth-of-discharge (DOD) of 191.3 Ah. Figure 1c,d show the temperature profiles for the charge and discharge processes, respectively. Because the 48TL200 module operates at high internal temperatures (265–350 °C), it is important to maintain the internal temperature of the module in a certain range to obtain optimum performance. As can be seen in Figure 1d, during the discharge process, the internal temperature of the module increases from 265 °C at the beginning of discharge or the EOC to 306 °C at EOD. In contrast to the discharge process, the temperature of the module decreases from 306 °C during the charge process (Figure 1c), and eventually the BMS turns on the internal heating system to keep the temperature of the module at 265 °C. It should be noted that battery module temperature increases during the discharge process, so an internal heating system is not required to maintain the operating temperature of 265 °C, as shown in Figure 1d. To understand the reason of the opposite behavior of the internal temperature for the charge and discharge processes, it is necessary to investigate the thermodynamics of the ZEBRA battery. In here, the internal temperature fluctuation will be briefly discussed along with the temperature profiles shown in Figure 1c,d. From the Gibbs-Helmholtz relation, the reaction free energy (ΔG) of Equation (1) is the utilizable electric energy of the battery and can be expressed as Equation (2):

∆G = ∆H − T·∆S(2)

where ∆H is the enthalpy change (theoretically available energy), T is the temperature, and ∆S is the entropy. The −T·∆S term shown in Equation (2) is often referred to as reversible heat (H_r_) that is released or absorbed during battery operation. A negative value of ∆S (−23.3 kJ/mol) has been reported for the discharge reaction in the literature, and it indicates that a positive amount of H_r_ is released during the battery discharge process [30]. In contrast to the discharge process, the battery will absorb the same amount of H_r_, which will be expressed as a negative amount of heat (−H_r_) released during the charge process. Therefore, the opposite temperature trend of the module shown for the charge and discharge processes (Figure 1c,d) can be briefly explained by the reversible heat generation or absorption. It should be mentioned that, in addition to H_r_ (reversible during the charge and discharge process), an irreversible heat generation process occurs for the module operation. This heat is known as Joule heat (H_j_) and is attributed to the battery ohmic resistance. Joule heat is proportional to the square of charge or discharge current and positive for both charge and discharge. The nearly linear decrease in temperature during charge shows this for the current range. Figure 1e shows the SOC profile of a typical module cycle consisting of the charge and discharge processes. The SOC decreases linearly for the discharge process as expected for constant discharge current. For the charge process, the SOC increases linearly during CC charging period, with the rate of SOC increase decreasing in CV charging mode due to the tapering of the charge current. Figure 1f shows that the SOC at EOC and EOD is unchanged for five cycles. The SOC at EOC and EOD are 200 Ah (full charged state) and 8.7 Ah. Overall, initial testing of the module shows stable capacity utilization of 95.7%. Efficiencies, such as Coulombic and energy efficiencies, are two significant parameters to determine the battery module effectiveness in energy-storage applications. Because of the use of BASE, which blocks material cross-over except for sodium-ion transport, the Coulombic efficiency of a ZEBRA battery is considered to be 100%. Therefore, only the energy efficiency of the module is calculated to evaluate the module performance in this work. To have more clear definition, energy efficiency is calculated for charge and discharge, battery, and the overall system. For the charge process, a battery cycler (NHR) provides energy to the BMS, which controls the battery module charging process and the miscellaneous energy loss, including the battery temperature control system (self-heating), the power to maintain the electronic operation of the BMS, etc. Charge efficiency (*E_c_*) can be calculated using Equation (3):

*E_c_* = *E_BC_*/*N_CE_* (3)

where *E_BC_* is the electric energy flowing into the battery pack and *N_CE_* represents the total energy exported from the NHR during the charge process. As one can see from Table 2, *E_BC_* and *N_CE_* are 10 kWh and 10.99 kWh for the initial testing cycle, respectively. Therefore, the calculated *E_c_* is 91.0% for the charge process.

In contrast to the charge process, the BMS is powered by the module itself during the discharge process. In other words, all miscellaneous energy consumption including self-heating and BMS operation is supplied by the module. The efficiency of discharge (*E_d_*) can be calculated using Equation (4).

*E_d_* = *N_DE_*/*E_BD_* (4)

During the discharge process, *N_DE_* and *E_BD_* are the amount of electric energy flowing back to NHR and the total discharge energy of the battery module, respectively. The *E_d_* of the discharge process can be as high as 98.8% because temperature increases during the discharge process, and the BMS is not required to run the internal heater at all. The only parasitic energy loss of the discharge process is the power consumption of the BMS electronics. To calculate the total energy efficiency for the cycle, beside *E_c_* and *E_d_*, it is necessary to calculate the energy efficiency of battery module (*E_b_*) itself.

*E_b_* = *E_BD_*/*E_BC_* (5)

If we use the values of *E_BD_* and *E_BC_* shown in Table 2 in Equation (5), the calculated *E_b_* is 90% for the battery module. It indicates that 90% of the electricity stored in the battery module can be reused and rest of the electricity (10%) is released as Joule energy (H_j_) that originates from the overall polarization of the battery module during the charge and discharge processes. The overall energy efficiency (*E_o_*) should be the combination of efficiencies from the charge process, battery, and discharge process of the battery module, and can be expressed as Equation (6).

∆G = ∆H − T·∆S*E_BD_*/*E_BC_* × *N_DE_*/*E_BD_* = *N_DE_*/*N_CE_* (6)

In fact, *E_o_* is the ratio of total energy returned to the NHR and the total energy exported from the NHR during a complete cycle of the battery module. Despite the need for internal heating to maintain the high operating temperature, as shown in Table 2, the *E_o_* of the battery module we tested can be as high as 80.9% for the initial cycles.

**Figure 2:**
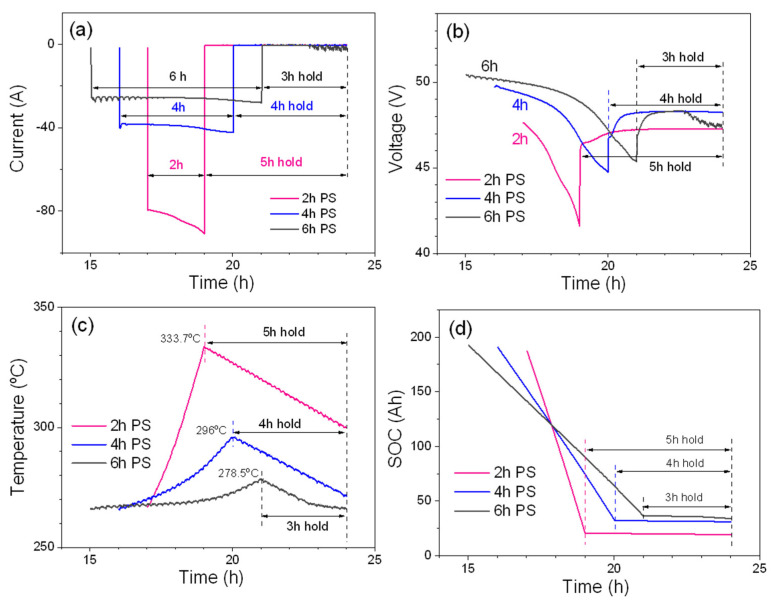
Peak-shaving test of 7.5 kWh discharge energy with the discharge time at 2, 4, and 6 h, respectively. (**a**) Current; (**b**) voltage; (**c**) temperature, and (**d**) SOC. An insert graph shown in (**d**) is a zoom-in view of SOC trends during the holding time.

Next, the battery module was tested under the schedule for peak-shaving applications. The fundamental idea of peak shaving is to shave peaks in load. Battery is charged during off-peak hours (when the electricity rate is low, or the amount of energy produced from renewable resources is abundant) and discharge the battery during peak hours of the day (when the electricity rate is high). As mentioned in the experimental section, the charging processes (CC + CV) of peak-shaving schedules are very similar; the main difference lies in each discharge time, such as 2, 4, and 6 h discharge, hereafter referred to as 2, 4, and 6 h peak shaving. Figure 2 shows consecutively current, voltage, temperature, and SOC profiles of 2, 4, and 6 h peak shaving by setting the total discharge energy to 7.5 kWh.

Because the total discharge energy is same for three peak-shaving tests, discharge currents for three peak-shaving tests also change accordingly. Average discharge currents in Figure 2a are ~85 A (~C/2.4 rate) for 2 h peak shaving, ~40 A (~C/5 rate) for 4 h peak shaving and ~26.5 A (C/7.5 rate) for 6 h peak shaving. As shown in Figure 2b, 2 h peak shaving shows lowest voltage across the discharge period due to the higher overpotentials. The EOD voltage is ~42.6 V for 2 h peak shaving, ~44.7 V for 4 h peak shaving and ~45.5 V for 6 h peak shaving, respectively. From the temperature graph shown in Figure 2c, one can see the temperature rises during discharge as mentioned in Figure 1d. The 2 h peak shaving has the highest temperature increase for the discharge process, then the 4 h discharge and 6 h discharge. This is in a good agreement with the order of discharge currents because 2 h peak shaving will generate the largest amount of H_j_, which is in addition to the H_r_, compared to that of the 4 h and 6 h peak-shaving periods. During the EOD-hold of 5 (2 h peak-shaving cycle), 4 (4 h peak-shaving cycle), and 3 h (6 h peak-shaving cycle), the module temperature will continuously decrease due to the heat loss through the insulation layers wrapped around the battery pack. It is worth mentioning that the module temperature does not fall below the operating temperature of 265 °C for the 2 h and 4 h peak-shaving tests. However, during hold times for the 3 h of 6 h peak-shaving test, the temperature falls below 265 °C and the self-heating needs to be turned on to maintain the operating temperature. The current fluctuation and the voltage drop (during the EOD-hold) of the 6 h peak shaving period shown in Figure 2a,b are good indication of initiating the self-heating system. Figure 2d shows the SOC levels during discharge and EOD-hold after discharge. As shown in Table 3, during discharge, the module discharges 166.3 Ah (83.2%) for the 2 h, 157.8 Ah (78.9%) for the 4 h, and 156.1 Ah (78.1%) for the 6 h peak-shaving periods, respectively. Capacity losses per h during the EOD-hold times follow 2 h peak-shaving period (0.28/h), 4 h peak-shaving period (0.3/h), and 6 h peak-shaving period (0.83/h). The large capacity loss for the 6 h peak-shaving period (the steeper slope indicated by an arrow in Figure 2d) agrees well with the faster battery drain via self-heating. The charge cycle is performed in a similar manner to the default cycle; that is, a CC charge at 40 A until the voltage reaches 53.4 V and then a CV charge to full capacity. The hold times after the EOC are approximately 5, 4 and 3 h for 2 h, 4 h, and 6 h peak-shaving periods, respectively. In contrast to the discharge process, the temperature of the module decreases and almost reaches a temperature of 265 °C during the charge process. Therefore, the internal heater needs to be turned on to maintain the operating temperature at 265 °C.

As shown in Table 3, after the EOC capacity reaches its full capacity at 200 Ah, the module slowly drains to supply power required to run the self-heating and BMS electronics during the EOC-hold period. It is not surprising to see a similar capacity loss per h (2.3–2.4 Ah/h) for EOC-hold times after charge for 2, 4, and 6 h peak-shaving tests because all the tests need to operate self-heating during EOC-hold periods to maintain the module at the operating temperature of 265 °C. Energy efficiencies for different peak-shaving tests are calculated by Equations (3)–(5) used for the full capacity testing shown above. The only difference is that peak-shaving tests have two holding periods after EOC and EOD. Therefore, when calculating the efficiency of charge and discharge for peak-shaving tests, it is important to consider the energy loss of the module during EOC-hold and EOD-hold as follows:

*E_o_* = *E_c_* × *E_b_* × *E_d_* = (*E_BC_*/*N_CE_*) × (*E_BD_*/*E_BC_*) × (*N_DE_*/*E_BD_*) = ((*E_f_* − *E_ch_*)/*N_CE_*) × (*E_BD_*/(*E_f_* − *E_ch_*)) × (*N_DE_*/*E_BD_*) (7)

Charge energy efficiencies for 6, 4, and 2 h peak-shaving periods are 81.1, 81.3, and 85%, respectively, which are significantly lower than *E_c_* (91.1%) of initial full cycling. This loss in *E_c_* mainly comes from the energy consumption for self-heating during EOC-hold period of the tests. In contrast to the charge process, module discharge energy efficiency (*E_d_*) for 6, 4, and 2 h peak-shaving tests are 98.3%, 99.1%, and 99.6%, respectively. Regardless of the duty cycle, *E_d_* is >98% for the module. As shown in Table 4, battery efficiency (*E_b_*) for 6, 4, and 2 h peak-shaving tests are 90%, 88.6% and 83.6%, respectively.

**Figure 3:**
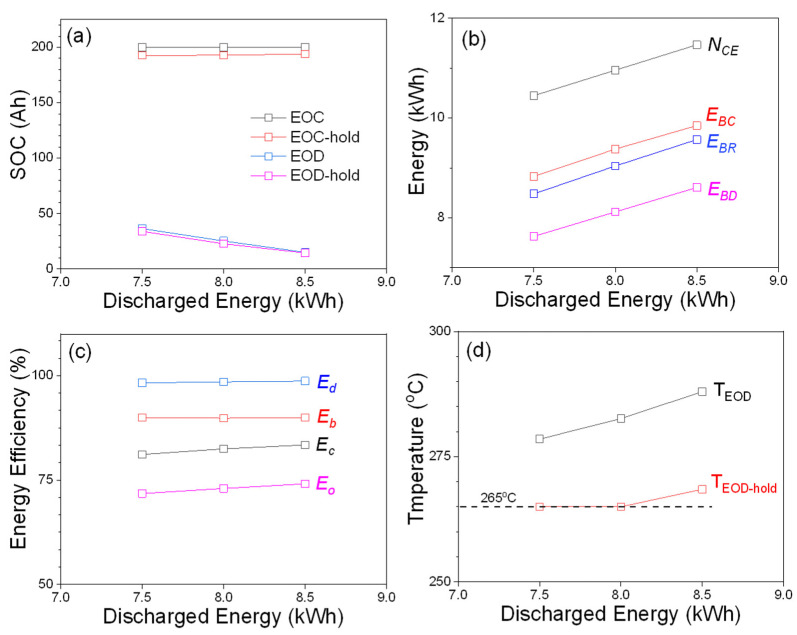
Module performances for peak-shaving tests with three different discharged energies: 7.5, 8.0, and 8.5 kWh. (**a**) SOC; (**b**) energy; (**c**) energy efficiency; (**d**) temperature of the module at EOD and EOD-hold.

The lower *E_b_* observed for the 2 h test (83.6%) can be attributed to its higher discharge current compared with that of 4 and 6 h tests. It is interesting to see that the overall efficiency is >70% at 7.5 kWh discharge energy for three typical duty cycles used for peak-shaving applications after considering all energy losses from operation of the self-heating and BMS electronics. The 6 h peak-shaving test was extended for higher discharged energies such as 8 and 8.5 kWh to further understand the capability of the module, and Figure 3 shows detailed comparisons for three 6 h tests. While increasing the discharged energy from 7 kWh to 8.5 kWh, the module needs to be discharged to lower SOCs to provide more energy.

As shown as Figure 3a, the SOCs at EOD are 25.3 Ah (12.6%) and 15.1 Ah (7.6%) for 6 h peak-shaving tests at 8 and 8.5 kWh, and these values show that the module has to discharge approximately 10 and 20 Ah more capacity compared to that at 7.5 kWh (36.5 Ah/18.3% at EOD). The energy plot in Figure 3b also shows a similar trend that the module utilizes more energy for the tests requiring higher discharged energy. Energy efficiencies are plotted in Figure 3c, and one noticeable advantage for peak-shaving tests with larger discharged energy is that *E_o_* increases from 71.8% (7.5 kWh) to 74.1% (8.5 kWh). This increased *E_o_* can be briefly explained from the temperature plot shown in Figure 3d. As can be seen, temperatures at EOD and EOD-hold of the 8.5 kWh test are 288 and 268.5 °C, respectively, which are higher than the minimum operating temperature of 265 °C. Therefore, self-heating will not be initiated for the entire EOD-hold and some part of the charge period until the temperature decreases to 265 °C. In contrast to the 8.5 kWh test, the module temperature reaches 265 °C during EOD-hold period for 7.5 and 8 kWh tests, and this triggers the self-heating that ultimately increases the electricity loss (lower *E_o_*) via heating. In general, the existence of rest steps for various PS tests will affect the overall efficiency of the module, because self-heating during the rest steps requires low power draining from the module itself.

**Figure 4:**
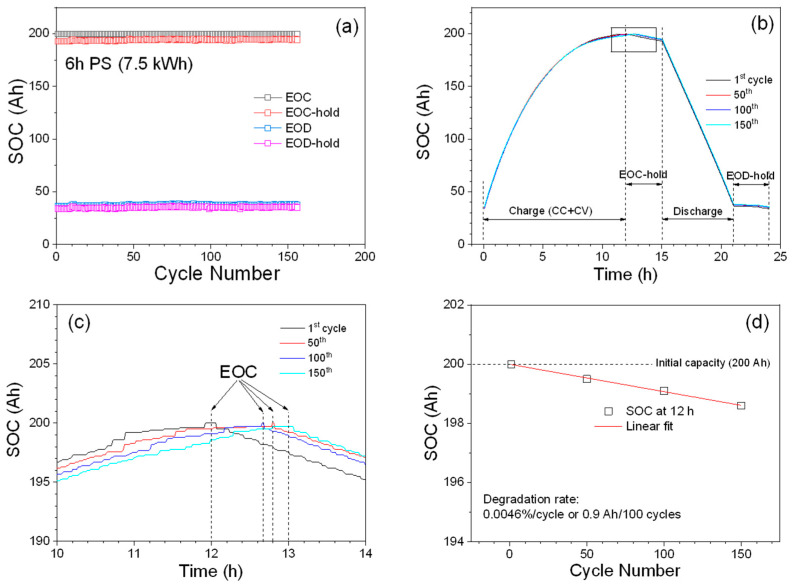
Long-term testing of the module under the 6 h peak-shaving schedule. (**a**) SOC vs. cycle; (**b**) comparison of SOCs for the 1st, 50th, 100th, and 150th cycles; (**c**) zoom-in SOC plots (area marked by a square in b) of the 1st, 50th, 100th, and 150th cycle; (**d**) capacity degradation rate for the charge.

To evaluate the long-term stability, the module was tested under the 6 h peak-shaving schedule over 150 cycles (150 days). Results from the initial examination (Figure 4a) show that SOCs at EOC, EOC-hold, EOD, and EOD-hold are quite stable over 150 cycles and further indicate that the strings and cells in the module run well according to the testing schedule without much deviation. SOC plots vs. time are shown in Figure 4b for four selected cycles (1st, 50th, 100th, and 150th), and SOC plots for the discharge and EOD-hold are quite consistent throughout 150 cycles. To see the detailed changes of the SOCs for the charge and EOC-hold, an enlarged SOC plot of the area marked with a square in Figure 4b is shown in Figure 4c. It can be seen that the charging time required for the first cycle is about 12 h and then slowly increases to about 13 h in the 150th cycle, which indicates that the battery material may be degraded and slowing down the charging process. To quantitatively describe the degradation rate of the charge process, we have measured the charge capacity after 12 h charging for the 1st, 50th, 100th, and 150th cycles, and made the linear fit shown in Figure 4d. If we assume that the slope of the linear fit can be used to represent the degradation of the charging process of the batteries in the module, the module degrades at a rate of 0.0046%/cycle (0.9 Ah/100 cycles), which indicates that the module can run for more than 4000 cycles before the module loses 20% of its initial capacity.

**Figure 5:**
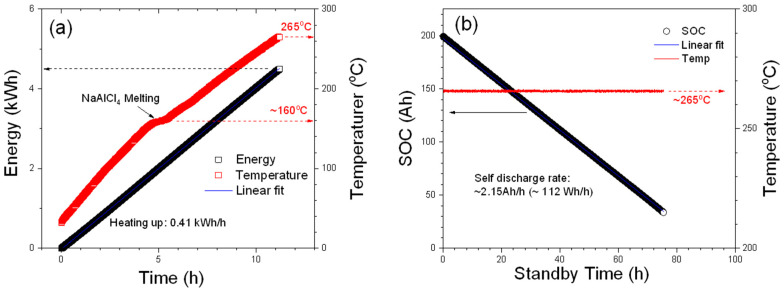
Initial heating-up and standby modes of the module. (**a**) Consumed energy and tem-perature of the heating-up process; (**b**) SOC and temperature of the module during the standby mode without getting power from external battery cycler.

Last but not least, the initial heating-up and the standby processes of the module were measured, and some detailed parameters are plotted in Figure 5.

When the module was initially heated up from the ambient temperature by the BMS, which is connected by the NHR system in this work, the average energy consumption of the initial heating-up process is 405 Wh/h that was obtained from the slope of the linear fit to the energy plot shown in Figure 5a The small plateau indicated by an arrow (near 160 °C) on the temperature plot can be attributed to the melting process of NaAlCl_4_, which is used as the secondary electrolyte in ZEBRA batteries and has a melting point at 157 °C. As shown in Figure 5b, the energy consumption during the standby mode is about 112 Wh/h (2.15 Ah/h), which is much lower than that of the heating-up process, and the module can sustain the operating temperature for more than 75 h just using the power from the battery pack before the module shuts off. It is worth mentioning that this module has experienced multiple intended and unintended shutdowns over the testing period of 1 year and has not shown any significant signs of malfunction from the thermal cycles leading to large temperature swings to the module and battery components.

## 4. Conclusions

In this paper, we describe detailed experimental studies conducted to evaluate the performance of a ZEBRA battery module for large-scale energy-storage applications. Operated at temperatures above 265 °C, the module can deliver an overall energy efficiency of 80.9% for a full capacity cycle consisting of a factory default charge (CC + CV) and a discharge with a 40 A discharging current. The battery efficiency calculated by considering only the energy absorbed and released from the module is 90%, which is much higher than the overall energy efficiency of the module, and this is mainly attributed to additional energy consumption for self-heating that is included to calculate the overall efficiency of the module. From the detailed analysis of energy efficiencies for each peak-shaving period, the energy efficiency of the discharge process (i.e., 98.8%) is much higher than that of the charge process (i.e., 91.1%). This finding agrees well with our observation that the energy loss from self-heating is much smaller for the discharge process because the temperature of the module rises from releasing both Joule and reversible heat. In contrast, the temperature of the charge process decreases and requires extensive application of self-heating to maintain the operating temperature of the module.

For the different peak-shaving duty cycles, the overall energy efficiencies of the module drop to 70.7–71.8% for 7.5 kWh energy utilization, and this can be attributed to the hold-time after the charge and discharge processes in the peak-shaving duty cycles. When energy utilization is increased from 7.5 kWh to 8.5 kWh, the overall energy efficiency increases from 71.8% to 74.1% because the involvement of self-heating (energy loss) is reduced for larger energy utilizations. Further, the long-term cycling of 6 h duty cycle shows quite stable trends for SOC and temperature. The capacity shows a degradation rate of 0.0046%/cycle over 150 cycles (150 days). The module also can be put into self-sustaining mode for over 70 h before most of the energy stored in the module is consumed via self-heating to maintain the operating temperature. In general, although self-heating is inevitable due to the high operating temperature, the ZEBRA module still shows quite reliable performance (e.g., high energy efficiency, slow degradation, etc.), and overall performance could be optimized by designing the parameters of the peak-shaving duty cycle in practical applications.

## Figures and Tables

**Table 1:** Technical Specification of 48TL200 Module.

Nominal Voltage, V	48
Module Energy, kWh	9.6
Module Capacity, Ah	200
Weight, lb.	231
Dimension, inch	19.5 (w) × 21.9 (d) × 12.6 (h)
Internal Temperature, °C	265–350
Environment Temperature, °C	−40–60

**Table 2:** Amount of energy and efficiencies of the charge and discharge processes.

NHR Charge Energy (*N_CE_*), kWh	10.99
Battery Charge Energy (*E_BC_*), kWh	10.0
Battery Discharge Energy (*E_BD_*), kWh	9.0
NHR Discharge Energy (*N_DE_*), kWh	8.89
Charge Energy Efficiency (*E_c_*), %	91.1
Discharge Energy Efficiency (*E_d_*), %	98.8
Battery Energy Efficiency (*E_b_*), %	90.0
Overall Energy Efficiency (*E_o_*), %	80.9

**Table 3:** SOC, energy and temperatures of the module for 7.5 kWh peak shaving test.

Discharge/Hold, h	6/3	4/4	2/5
Discharge Energy, kWh	7.5	7.5	7.5
EOC, Ah	200	200	200
EOC-Hold, Ah	192.5	190.8	187
Cap. Loss EOC-Hold, Ah	7.2 (2.4/h)	9.2 (2.33/h)	11.6 (2.32/h)
EOD, Ah	36.4	33	20.7
Discharge Capacity, Ah/DOD, %	156.1/78.1	157.8/78.9	166.3/83.2
Temp. at EOD, °C	278.5	296	333.7
EOD-Hold, Ah	34.0	31.1	19.3
Temp. at EOD-Hold, °C	265	271.4	300
Cap. Loss EOD-Hold, Ah	2.5 (0.83/h)	1.2 (0.3/h)	1.4 (0.28/h)

**Table 4:** Energy efficiency calculation for 7.5 kWh peak shaving tests.

Discharge/Hold (h)	6/3	4/4	2/5
NHR Discharge Power, kW	1.25	1.875	3.75
*E_c_* %	81.1	81.3	85.0
*E_b_* %	90.0	88.6	83.6
*E_d_* %	98.3	99.1	99.6
*E_o_* %	71.8	71.4	70.7

## Data Availability

Data is contained within the article.
